# Effect of Exogenous Application of Nicotinic Acid on Morpho-Physiological Characteristics of *Hordeum vulgare* L. under Water Stress

**DOI:** 10.3390/plants11182443

**Published:** 2022-09-19

**Authors:** Taimoor Hassan Farooq, Muhammad Adnan Bukhari, Muhammad Shahid Irfan, Muhammad Rafay, Awais Shakoor, Muhammad Haroon U. Rashid, Yang Lin, Muhammad Saqib, Zaffar Malik, Nouman Khurshid

**Affiliations:** 1 Bangor College China, A Joint Unit of Bangor University and Central South University of Forestry and Technology, Changsha 410004, China; 2 Department of Agronomy, Faculty of Agriculture and Environment, The Islamia University of Bahawalpur, Bahawalpur 63100, Pakistan; 3 Department of Forestry, Range and Wildlife Management, Faculty of Agriculture and Environment, The Islamia University of Bahawalpur, Bahawalpur 63100, Pakistan; 4 Teagasc, Environment, Soils and Land Use Department, Johnstown Castle, Co., Y35 Y521 Wexford, Ireland; 5 Department of Forestry and Range Management, University of Agriculture, Faisalabad 38000, Pakistan; 6 Department of Soil Science, Faculty of Agriculture and Environment, The Islamia University of Bahawalpur, Bahawalpur 63100, Pakistan

**Keywords:** growth, yield, anatomical attributes, physicochemical characters, barley, water deficit

## Abstract

Abiotic stresses, such as high temperature and drought conditions, greatly influence the development of plants and the quality and quantity of products. Barley (*Hordeum vulgare* L.) crop production is largely impacted by drought, affecting growth, yield, and ultimately the productivity of the crop in hot arid/semi-arid conditions. The current pot experiment was directed to observe the outcome of nicotinic acid (NA) treatments on barley’s physiological, biochemical, and production attributes at two capacity levels, i.e., 100% normal range and withholding water stress. Randomized complete block design (RCBD) was used during the experimentation with the two-factor factorial arrangement. NA was applied exogenously by two different methods, i.e., foliar and soil application (fertigation). NA solution contained various application levels, such as T1 = control, foliar applications (T2 = 0.7368 gL^−1^, T3 = 1.477 gL^−1^, T4 = 2.2159 gL^−1^), and soil applications (T5 = 0.4924 gL^−1^, T6 = 0.9848 gL^−1^, and T7 = 1.4773 gL^−1^). Results depicted that, overall, foliar treatments showed better effects than control and soil treatments. Plant growth was preeminent under T4 treatment, such as plant height (71.07 cm), relative water content (84.0%), leaf water potential (39.73-MPa), leaf area index (36.53 cm^2^), biological yield (15.10 kgha^−1^), grain yield (14.40 kgha^−1^), harvest index (57.70%), catalase (1.54 mmolg^−1^FW^−1^), peroxidase (1.90 g^−1^FWmin^−1^), and superoxide dismutase (52.60 µgFW^−1^) were superior under T4 treatment. Soil plant analysis development (54.13 µgcm^−2^) value was also higher under T4 treatment and lowest under T7 treatment. In conclusion, NA-treated plants were more successful in maintaining growth attributes than non-treated plants; therefore, the NA foliar treatment at the rate of 2.2159 gL^−1^ is suggested to find economical crop yield under drought conditions. The present study would contribute significantly to improving the drought tolerance potential of barley through exogenous NA supply in water deficit areas.

## 1. Introduction

Drastic climate changes and increased water scarcity challenge global food security, which is further exacerbated due to the need to feed a growing global population. Global agricultural production might need to increase by 60–110% to meet the increasing demands of food security. Barley (*Hordeum vulgare* L.) is a tough grain crop of significant importance worldwide, grown in several environments where other grains cannot grow [1]. It has broad flexibility to various agro-climatic circumstances and distinctive soil attributes [2]. It is utilized as food for innumerable creatures. Its success is due to its capacity to produce in unfavorable atmospheres, such as dry seasons, low temperatures, and salt [3]. This grain is the fourth biggest nutrition crop. Among the primary oats, its global total grain planting area is 47 million hectares, and the annual output is 147.4 million tons, whereas the typical efficiency is 3136 kgha^−1^ [1]. Moreover, the yield of winter grain is 19–33% higher than that in spring, which expands the financial aspects of the harvest [4].

Nicotinic acid (NA) is a fundamental water-solvent plant nutrient. It normally exists in two structures, niacin, and nicotine amide [5]. NA can be obtained through both endogenous and exogenous sources. NA is a precursor to the co-enzymes nicotinamide adenine dinucleotide (NAD+) and nicotinamide adenine dinucleotide phosphate. NAD+ and NADP+ are important co-enzymes involved in various electron transport measurements [6,7]. Energy saves prompted by NA are utilized for tissue arrangement at various formative stages [8,9]. This reality was affirmed by using NA and thiamine to lessen the unsafe impacts of multiple pressures throughout the sprouting and starting advancement phases of plants [10].

Abiotic stresses, such as high temperature, salt formulations [11,12], inorganic treatments [13,14], toxicity [15], pollution stress [16], and drought conditions [17], greatly interfere with the development of plants, and the quality and quantity of products [18,19]. Sufficient internal water is fundamental for ideal seedling germination, and subsequent growth as water is the primary factor associated with germination [20]. Water pressure affects plant development, diminishing dry matter aggregation, subsequently limiting the pace of photosynthesis, and lessening the assimilation of fundamental supplements for plants [21]. Using natural added substances can lessen the adverse consequences on plant development by working on plants’ physical and compound properties [22].

Environmental changes and water shortages are global threats to agriculture. Water scarcity is the main obstacle to obtaining viable agronomic crop production. To overcome unfavorable agro-climatic conditions and sustainable agriculture, new agronomic practices and economical and easy-to-operate farming methods are the need for modern agriculture. Therefore, this study’s objective is to examine the barley’s response to water stress under foliar and soil exogenous treatments of nicotinic acid. This project means to upgrade the physiological and biochemical qualities of grain crops and work on the resistance of grain crops under affliction conditions.

## 2. Materials and Methods

### 2.1. Experimental Site

The trial was conducted in the greenhouse of the Department of Agronomy, University College of Agriculture, Islamia University Bahawalpur. Pots (25 × 15 cm) filled with 7.5 kg of sterilized soil were used. Ten healthy and vigorous barley seeds per pot were selected randomly and sown in pots at 6 cm depth, keeping the same distance among seeds. This experiment was carried out under control conditions (100% FC) and withholding stress. A completely randomized factorial design (CRD) was used with six repetitions. Soil analysis was performed following the standard methods for determining physicochemical parameters. Soil physiochemical properties are shown in (Table 1).

### 2.2. Experimental Details

The germ-plasm of barley was collected from Regional Agricultural Research Institute (RARI) Bahawalpur. Nicotinic acid (NA) was applied via foliar and soil applications under well-watered levels (FC 100%) and water deficit conditions. Treatment levels are: T1: control, foliar application treatments (T2: NA@0.7368 gL^−1^, T3: NA@1.477 gL^−1^, T4: NA@2.219 gL^−1^), and soil application treatments (T5: NA@0.4924 gL^−1^, T2: NA@0.9848 gL^−1^, T2: NA@1.4773 gL^−1^). 

The barley plants were harvested for analysis after 106 days (almost 3.5 months). After harvesting, three replicates per treatment were selected for further analysis. 

### 2.3. Morphological Parameters and Root Plasticity

Plant roots were separated from shoots after thoroughly cleaning them with distilled water. Roots and shoot lengths were measured using a scale, and their fresh and dry weights were recorded to calculate the crop growth rate. For plant dry weight, specimens are dried in an oven at 75 °C. Calculations were made based on the shoot and root dry. Fresh roots were used to calculate root surface area (cm^2^), root volume (cm^3^), and root tips using root scanners (Win RHIZO Pro, STD, 2017, Regent Instruments Inc., Quebec City, QC, Canada). Root diameter (mm) was calculated with a microscope’s help by manually removing soil particles from roots until all the roots were cleared from the soil particles.

### 2.4. Yield Parameters

Spike length (cm) was calculated with the help of measuring tape at the time of fully matured barley plants. The number of spikelets per spike was counted by selecting six spikes randomly from the top and bottom. The number of tillers per plant and the number of grains per spike were calculated manually, and average data were used for the description of the results.

The weight of 1000 grains (g) from the individual pots of each replication was measured by electric balance. To determine the biological yield per plant (kgha^−1^), plants were selected randomly from each treatment and carefully picked and splashed with water to eliminate soil particles, later sun-dried. The plants were weighed to record the biological yield. For the calculation of grain yield (kgha^−1^), the one-meter square was selected, and the number of heads was counted. This was repeated 5 times to get the average, and the yield was measured using the following formula.

Yield in t/ha = (A × B × C)/10,000

The subsequent formula was used to analyze barley plants’ harvest index (%).

$$HI=\frac{Grain\;Yield}{Biological\;yield}\times 100$$

### 2.5. Physiological Parameters

Leaf area index (cm^2^) was measured on the plants’ fresh, fully developed leaves per pot. The average leaf area of barley was measured by using an instrument planimeter. To calculate the SPAD value of the leaves, the SPAD instrument was used (Minolta, Osaka, Japan). Three leaves of fully developed pots were measured. For the determination of water potential (-Mpa), three leaves were harvested from the top portion of barley plants. The water potential was measured by a Scholander pressure chamber. Leaf samples were taken from three treatment plants to determine relative water content (%). The digital balance was used to take each plant sample’s fresh weight (FW) and was soaked in distilled water in test tubes. The moisture level was removed with tissue paper for the turgid weight (TW), and samples were placed in the oven at a temperature (65 °C) for 72 h to take the dry weight (DW). The relative water content (RWC) was measured using the following equation described by [23].

$$RWC\;\left(\%\right)=\frac{\left(FW-DW\right)}{\left(TW-DW\right)}\times 100$$

Whereas

DW=Dry weight, FW=Fresh weight, TW=Turgid weight

A spectrophotometer was used to evaluate the antioxidant enzyme’s activity (Hitachi-2800). Barley leaves were homogenized in 50 mM phosphate buffer with pH of 7.0 and 1 mM dithiothreitol (DTT) to measure peroxidase (POD), superoxide dismutase (SOD), and catalase (CAT), based on information provided [24,25]. A calculation was made to determine the enzymatic activity by measuring the amount of hydrogen peroxide converted to hydrogen and water molecules [25]. This enzyme was evaluated in a 3 mL solution that included a 50 mM buffer solution with a 7.0 pH, 5.9 mM hydrogen peroxide extract, and 0.1 mL enzyme. The consumption of hydrogen peroxide determined the CAT activity by observing the reduction in the transmission density of light at 240 nm every 20 s. The change in absorbance was measured as a single unit CAT activity. The oxidation of hydrogen peroxide as an electron donor with guaiac was used to calculate POD. A mixture was prepared for peroxidase analysis (POD) comprising a phosphate buffer 50 mM, 5.0 pH, guaiacol 20 mM, hydrogen peroxide 40 mM, and enzyme 0.1 mL. Production of tetraguayacol at 470 nm caused an increase in light absorption via a solution, which was investigated. The quantity of enzyme responsible for the 0.01 rise in optical density value in 1 min was one unit. The enzyme’s activity was calculated at one unit per minute per gram of fresh weight. SOD was measured by the following method [26]. For photochemical examination of nitroblue tetrazolium (NBT), 0.2 g of the sample was standardized in one percent polyvinylpyrrolidone (PVP) 50 mM, potassium phosphate buffer 7, pH 7.0, centrifuge at 15,000 rpm at 40 °C for 30 min. Translucent supernatant 0.5 mL, methylene tetra-acetic acid 2 mL, methionine 20 mM, NBT 0.12 mM, micromolar riboflavin 0.5 mL, and PVP 0.5 mL were used to make the reaction mixture. Observations were conducted calorimetrically against the blank at 560 nm optical density.

### 2.6. Statistical Analysis

Recorded observations on desired attributes from the current experimental trial and means of recorded data from every pot in each repeat were statistically evaluated. A two-way analysis of variance (ANOVA) technique was used for data analyses to understand the impact of NA and water stress treatments. Significant differences between the treatment means were compared using the least significant difference test (LSD) at 5% probability levels among all treatments. All statistical analyses were performed using the SPSS Statistical Package (SPSS 17.0, IBM, Chicago, IL, USA).

## 3. Results

### 3.1. Plant Morphological Attributes

#### 3.1.1. Plant Height (PH) and Root Length (RL)

**Figure 1:**
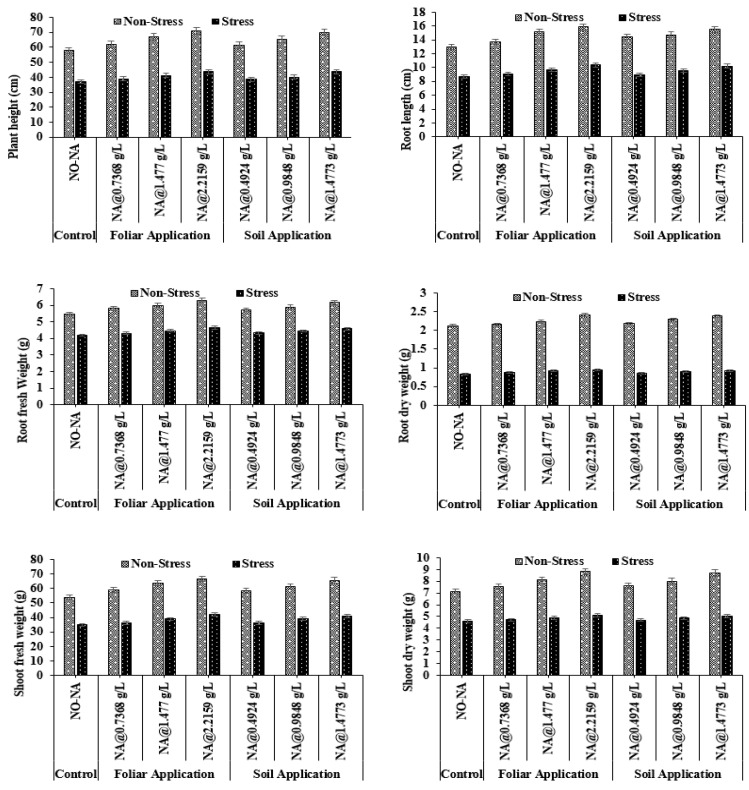
Effect of exogenously applied nicotinic acid (NA) on barley plant height (cm), root length (cm), root fresh weight (g), root dry weight (g), shoot fresh weight (g), and shoot dry weight (g) at anthesis stage of plant under control and water deficit stress levels.

The effect of different doses of NA on PH and RL was found to be significant using statistical analysis (*p* = 0.041 for PH and *p* = 0.034 for RL). The highest PH and RL were recorded under treatment T4 (71.07 cm and 15.87 cm, respectively), while treatment T5 recorded the lowest values (57.97 cm and 13.01 cm, respectively) at 100% FC. Under water deficit conditions, maximum values of PH and RL (43.73 cm and 10.43 cm, respectively) were observed under T7, and minimum values (36.90 cm and 87.77 cm, respectively) were observed under T1 (Figure 1). The interaction of NA and water stress treatments was found to be non-significant for both plant height and root length (*p* > 0.05 for both) (Figure 1).

#### 3.1.2. Root Fresh and Dry Biomass

Treatment T4 yielded the highest values of root fresh and dry weight (6.31 g and 2.41 g, respectively), while treatment T5 yielded the lowest (5.49 g and 2.14 g, respectively) at 100 percent FC. Under water deficit conditions, the maximum values of root fresh and dry weight (4.66 g and 0.95 g, respectively) were observed in T7, and minimum values (4.20 g and 0.84 g, respectively) were observed in T1. The interaction of NA and water stress treatments was found to be non-significant for fresh root weight (*p* = 0.058), whereas it was significant for root dry weight (*p* = 0.043) (Figure 1).

#### 3.1.3. Shoot Fresh and Dry Biomass

Maximum values of shoot fresh and dry weight (66.67 g and 8.82 g, respectively) were observed under T4 treatment, whereas minimum (54.07 g and 7.11 g, respectively) were recorded in treatment under T5. Under water deficit conditions, maximum values (42.00 g and 5.11 g, respectively) were observed under T7, and minimum values (34.87 g and 4.62, respectively) were observed under T1. The interaction of NA and water stress treatments was found to be non-significant for fresh shoot weight (*p* = 0.064), whereas, for root dry weight, it was significant (*p* = 0.038) (Figure 1).

#### 3.1.4. Spikes Count and Spike Length

**Figure 2:**
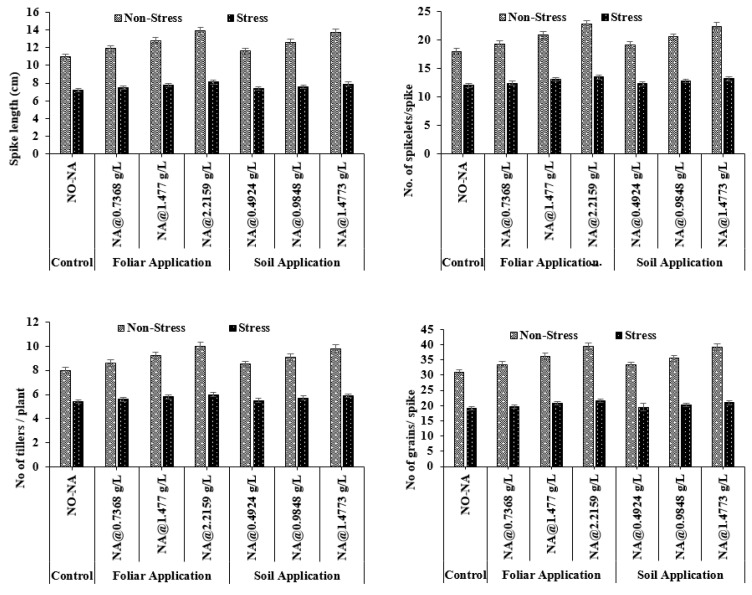
Effect of exogenously applied nicotinic acid on spike length (cm), spikes count per plant, tiller count per plant, and number of grains at anthesis stage of the plant under control and water deficit stress levels.

Among all treatments, the maximum spikes per plant and spike length (22.40 cm and 13.90 cm, respectively) were observed in the T4 treatment, and a minimum (18.03 and 11.03 cm, respectively) was recorded in the T5 treatment. Under water deficit conditions, maximum spikes per plant and spike length (13.50 and 8.10 cm, respectively) were observed under T7 treatment, and minimum values (12.03 and 7.23 cm, respectively) were observed under T1. The interaction between treatments was significant for both spikes count and spike length (*p* = 0.024) (Figure 2). 

#### 3.1.5. Number of Tillers and Grains Per Plant

The maximum average value of tillers and grains per plant (10.33 and 39.40, respectively) was observed in the T4 treatment, whereas a minimum (8.03 and 28.10, respectively) was recorded in treatment T4. Under water deficit conditions, maximum values of tillers and grains per plant (6.07 and 20.50, respectively) were observed in the T7 treatment, and minimum values (5.40 and 17.83) were observed in T1. The interaction between treatments was significant for parameters (*p* = 0.046) (Figure 2).

### 3.2. Root Plasticity

#### 3.2.1. Root Surface Area and Root Volume

**Figure 3:**
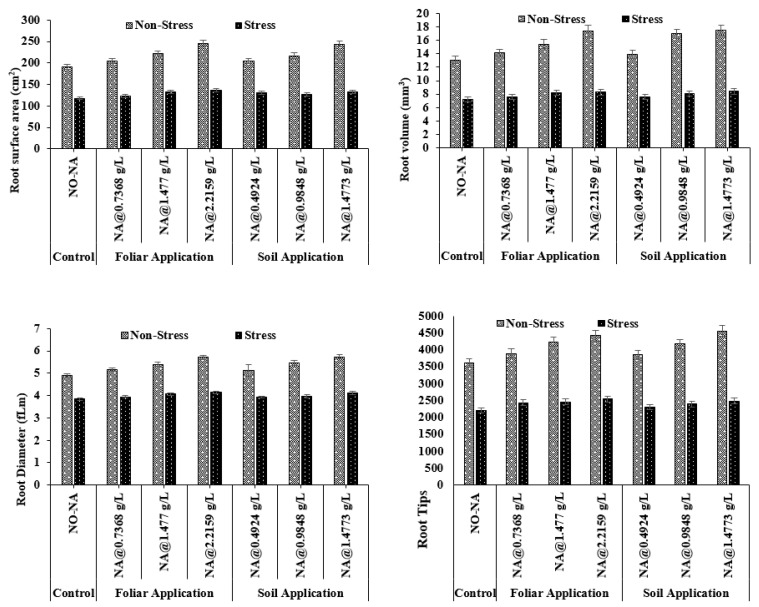
Effect of exogenously applied nicotinic acid on barley root surface area (cm^2^), root volume (cm^3^), root diameter (mm), and root tips at anthesis stage of plant under control and water deficit stress levels.

The maximum value of root surface area (246.00 cm^2^) was observed under the T4 treatment, whereas the minimum (191.17 cm^2^) was recorded under the T5 treatment. Under water deficit conditions, a maximum value (136.04 cm^2^) was observed in T7 (NA@1.4773 gL^−1^), and a minimum value (118.33 cm^2^) was observed in T1 (control). The interaction between NA and water stress treatments was significant (*p* = 0.038) (Figure 3).

The maximum value of root volume (19.57 cm^3^) was observed under the T4 treatment, whereas the minimum (15.13 cm^3^) was recorded under T5. Under water deficit conditions, a maximum value (10.50 cm^3^) was observed under T7, and a minimum value (9.19 cm^3^) was observed in T1. The interaction between NA and water stress treatments was significant (*p* = 0.029) (Figure 3).

#### 3.2.2. Root Diameter and Root Tips

The maximum values of root diameter and root tips (4.74 mm and 4577.1, respectively) were observed under T4, whereas minimum (3.93 mm and 3610.7, respectively) were recorded in T5. Under water deficit conditions, maximum values of root diameter and root tips (3.98 mm and 2542.1) were observed in T7, and minimum values (2.85 mm and 2207.1) were observed in T1. The interaction of NA and water stress treatments was significant for root diameter (*p* = 0.019), whereas it was non-significant for root tips (*p* = 0.053) (Figure 3).

### 3.3. Plant Leaf Dynamics

#### 3.3.1. Leaf Area Index (LAI)

**Figure 4:**
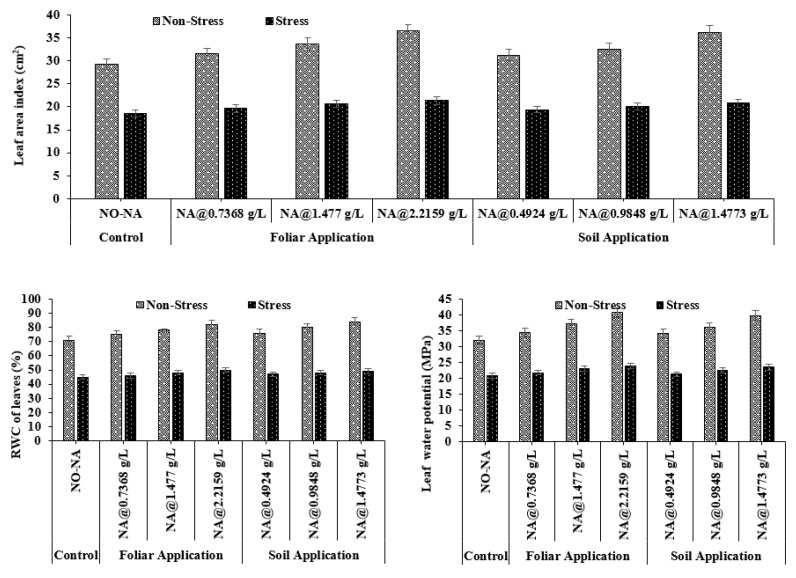
Effect of exogenously applied nicotinic acid on leaf area index (cm^2^), the relative water content of leaves (%) and leaf water potential (MPa) at anthesis stage of plant under control and water deficit stress levels.

Overall, the maximum value of LAI (36.53 cm^2^) was observed in T4, whereas a minimum (29.20 cm^2^) was recorded in treatment (NA@0.4924 gL^−1^) at 100% FC. Under water deficit conditions, a maximum value (21.40 cm^2^) was observed in T7, and a minimum value (18.67 cm^2^) was observed in T1. The interaction between NA and water stress treatments was non-significant (*p* = 0.047) (Figure 4). 

#### 3.3.2. Relative Water Content (RWC) and Leaf Water Potential

Among all treatments, the maximum values of RWC and leaf water potential (84.0% and 39.73-MPa, respectively) were observed in T4 and minimum (71.0% and 31.00-MPa, respectively) in treatment under T5 treatment. Under water deficit conditions, maximum values of RWC and leaf water potential (50.07% and 22.90-MPa) were observed in T7, and minimum values (45.07% and 19.00-MPa) were observed in T1. The interaction of NA and water stress treatments was found to be non-significant for RWC and leaf water potential (*p* > 0.05 for both) (Figure 4). 

### 3.4. Plant Enzymatic Responses

**Figure 5:**
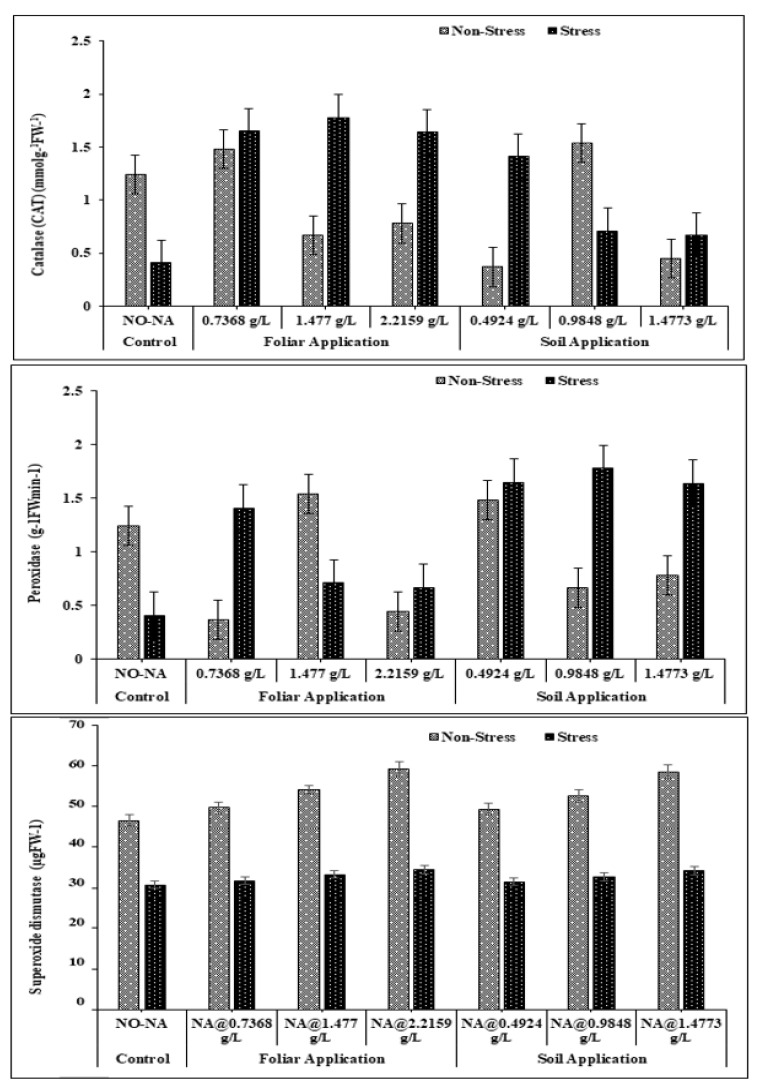
Effect of exogenously applied nicotinic acid on catalase (CAT) (mmolg^−1^FW^−1^), peroxidase (POD) g^−1^FWmin^−1^), superoxide dismutase (SOD) at anthesis stage of the plant under control and water deficit stress levels.

The maximum values of CAT, POD, and SOD (1.54 mmolg^−1^FW^−1^, 1.90 g^−1^FWmin^−1^, 52.60 µgFW^−1^, respectively) were observed in T4, whereas minimum values were recorded in treatment T5 at 100% FC. Under water deficit conditions, maximum values of CAT, POD, and SOD (1.78 mmolg^−1^FW^−1^, 1.40 FWmin^−1^, 33.20 µgFW^−1^, respectively) were observed under the T7 treatment and minimum value (0.41 mmolg^−1^FW^−1^, 0.20 g^−1^FWmin^−1^, 28.80 µgFW^−1^, respectively) were observed in T1. The interaction of NA and water stress treatments was significant (*p* < 0.05) for all the enzymes (Figure 5). 

### 3.5. Soil Plant Analysis Development (SPAD)

**Figure 6:**
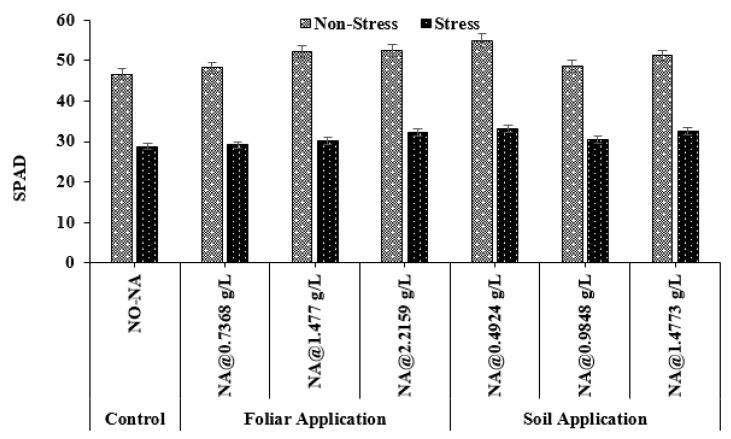
Effect of exogenously applied nicotinic acid on Soil Plant Analysis Development (SPAD) at the anthesis stage of the plant under control and water deficit stress levels.

The Soil Plant Analysis Development (SPAD) is one of the most commonly used diagnostic tools to measure crop nitrogen status. Statistical analysis for the SPAD data value showed a significant effect of different doses of NA. The maximum SPAD value (54.13) was observed in T4, whereas the minimum (45.70) was recorded in the T5 treatment. Under water deficit conditions, a maximum value (32.17) was observed in T7 and a minimum value (27.83) was observed in T1 (control). The interaction between NA and water stress treatments was significant (*p* = 0.041) (Figure 6).

### 3.6. Yield Parameters

#### 3.6.1. 1000-Grain’s Weight (g)

**Figure 7:**
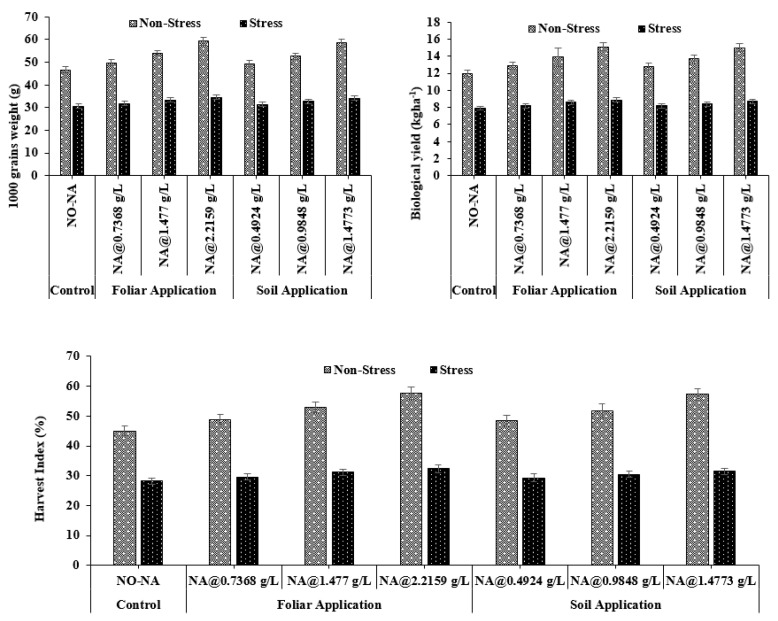
Effect of exogenously applied nicotinic acid on thousand-grain weight (g), grain yield (Kg ha^−1^), and harvest index at anthesis stage of plant under control and water deficit stress levels.

Overall, a maximum value of 10,000-grain weight (58.20 g) was observed in the T4 treatment, whereas a minimum (45.70 g) was recorded in treatment T5, at 100% FC. Under water deficit conditions, a maximum value (34.51 g) was observed in T7, and a minimum value (30.71 g) was observed in T1. The interaction between NA and water stress treatments was non-significant (*p* = 0.057) (Figure 7).

#### 3.6.2. Grain Yield

The maximum value of grain yield (15.10 kgha^−1^) was observed in T4, whereas the minimum (12.00 kgha^−1^) was recorded in treatment T7 at 100 % FC. Under water deficit conditions, a maximum value (8.9 kgha^−1^) was observed in T7, and a minimum value (7.5 kgha^−1^) was observed in T1. The interaction between NA and water stress treatments was significant (*p* = 0.046) (Figure 7).

#### 3.6.3. Harvest Index

The maximum value of harvest index (57.70%) was observed in T4, whereas the minimum (45.00%) was recorded in treatment T5 at 100 % FC. Under water deficit conditions, a maximum value (32.30%) was observed in T7, and a minimum value (28.20%) was observed in T1. The interaction between NA and water stress treatments was significant (*p* = 0.028) (Figure 7).

## 4. Discussion and Conclusions

Usually, drought stress causes plant growth retardation, reducing plant growth and yield [27,28]. In the current experiment, the barley’s root biomass, root and shoot length were reduced significantly under water-stressed conditions. Severe drought stress applied before the anthesis stage significantly affected barley growth as the water stress level increased, root volume was reduced, causing variations in the root and shoot ratio. Barley biomass accumulation was reduced by 57% under severe drought stress. The possible reason is drought can cause nutrient deficiencies, even in fertilized soils, due to the reduced mobility and absorbance of individual nutrients, leading to a retarded growth [21,22,23]. Moreover, unbalanced fertilizer use, and other unhealthy agronomic practices have seriously led to the root cause of crop growth and yield decline [29]. Zhang et al. also mentioned that drought significantly decreased the agronomic traits of wheat and rice with biomass and yield showing the largest decreases. Drought decreased wheat biomass and yield by 25.0% and 27.5%, respectively, and decreased rice biomass and yield by 25.2% and 25.4%, respectively. Moreover, wheat grown in pots showed greater decreases in agronomic traits than those grown in the field [30]. 

The number of spikelets per spike is the most sensitive parameter in plants that are affected by moisture stress during the reproductive stage. Water stress affects tiller formation and reduces the possibility of tillers transforming into spikes, resulting in spike abortion [31]. It might be due to moisture deficiency during the flowering and grain development stages causing a significant loss leading to serious abscission of tillers and photosynthetic absorption, consequently reducing yield [32]. Another important factor regarding growth and yield is 1000-grain weight; in this study, 1000-grain weight was substantially reduced when the plants were treated with water stress. The possible reason is that the plant produces smaller seeds due to moisture stress during the seed filling stage [32,33]. 

In this experiment, a water deficit stress negatively impacted relative water content. Compared to the control condition, plants were affected by drought stress, thus causing low water potential, SPAD value, and LAI. The results are similar to [34], who suggested that plant leaves lose water potential due to water scarcity. Another study also reported that under water deficit conditions, triticale genotypes had higher negative osmotic potential [35]. Furthermore, the chlorophyll content in barley leaves was suppressed under drought stress [33]. Barley grain yield under drought stress conditions was negatively correlated with leaf water potential [33]. By adjusting osmotic potential, plants can maintain their turgor pressure and high water contents inside the cells under drought-stress conditions [36]. It is reported that the decrease in LAI and photosynthetic activity due to water deficit stress would decrease biological yield [22]. The decline in economic yield due to water stress is the shortening of plant growth and grain filling time [37]. By decreasing unnecessary transpiration, NA improves water use efficiency, resulting in greater economic production. In our study, we found similar results that describe a decrease in economic yield, total biomass, and harvest index in barley plants exposed to water stress [10,22].

The enzymatic antioxidant (CAT, SOD, and POD) and contents of carotenoids, α- tocopherols, and ascorbic acid flavonoids, are non-enzymatic antioxidants involved in the defense mechanism of plants. In plants, oxidative stress is often induced generally due to the closing of stomata under water deficit conditions, indicating a reduction in photosynthetic activity [38]. It causes damage to the proper working of plants by causing oxidative damage to proteins, fats, nucleic acid, and enzymes [39]. Our study showed an increase in CAT, SOD, and POD activity after the NA application in barley plants exposed to water deficit stress. More or less similar results regarding physiological activities increment due to inorganic treatment applications compared to water deficit stress were observed by many researchers in different crops [39,40,41].

Drought has been one of the most important limiting factors for crop production, which deleteriously affects food security worldwide. It will be compulsory to increase productivity and economic yield under conditions constrained by water availability. However, before this can occur, the extent of crop reduction and other agronomic traits that are affected by changes in the climate must be understood. The application of NA helps to improve the number of tillers per plant and the number of spikelets per spike, which play a key role in the grain yield of wheat crops. When NA was applied at the rate of 2.2159 gL^−1^ as foliar treatment and 1.47732 gL^−1^ as a soil application treatment under water deficient stress levels, it increases plant height, leaf area, and the number of leaves, which plays a role in stomata opening, which increases stomatal conductance, leaf water potential and relative water content, and improves cell wall integration. NA improves enzymatic activities and increases photosynthetic and transpiration rates, which help get the maximum yield of the wheat crop. This study was performed as a pot experiment, therefore, NA applications in field conditions need to be tested under diversified stress conditions on various crops in Pakistan. The ultimate objective is to make this technique cost-effective and economically practicable for farmers to achieve maximum output.

## Figures and Tables

**Table 1:** Soil physiochemical properties before sowing of barley seeds.

Soil Properties	Range
pH	7.54
Electrical conductivity (EC)	0.02
Organic matter %	0.61
EC (electrical conductivity, (dsm^−1^ at 250 °C)	3.18
Available phosphorus (ppm)	8.1
Available potassium (ppm)	136
Soil color	Reddish brown
Texture of soil	Sandy loam

## Data Availability

All the available data have been presented in the manuscript.
